# Effect of Heat Treatments on the Corrosion Resistance of a TRIP Steel and Its Evaluation by Non-Destructive Testing

**DOI:** 10.3390/ma19132728

**Published:** 2026-06-25

**Authors:** Karla Ivette Vega-Nava, Ariosto Medina-Flores, Marco Antonio Espinosa-Medina, José Sergio Pacheco-Cedeño, Héctor Guillermo Carreón-Garcidueñas, Francisco Fernando Curiel-López, José Jaime Taha-Tijerina

**Affiliations:** 1 Instituto de Investigación en Metalurgia y Materiales, Universidad Michoacana de San Nicolás de Hidalgo, Morelia 58000, Mexico; 1234584b@umich.mx (K.I.V.-N.); hcarreon@umich.mx (H.G.C.-G.); francisco.curiel@umich.mx (F.F.C.-L.); 2 Facultad de Ingeniería Mecánica, Universidad Michoacana de San Nicolás de Hidalgo, Morelia 58000, Mexico; marco.espinosa@umich.mx; 3 Tecnológico Nacional de México, Instituto Tecnológico de Morelia, Morelia 58120, Mexico; spacheco_isa@yahoo.com.mx; 4 Mechanical Engineering Department, The University of Texas Rio Grande Valley, Brownsville, TX 78520, USA

**Keywords:** TRIP Steels, microstructure, corrosion, austenite phase, electrical conductivity, hardness, eddy current testing, microstructural homogeneity

## Abstract

**Highlights:**

**What are the main findings?**

- Full austenitization increased retained austenite to 12.7%.
- Route A reduced corrosion current density by up to 54%.
- Heat treatment improved corrosion resistance through microstructural refinement.
- Eddy current measurements correlated with electrochemical behavior.

**What are the implications of the main findings?**

- Retained austenite promotes enhanced corrosion resistance.
- Optimized heat treatment improves TRIP steel durability.
- Eddy current testing enables predictive corrosion assessment.
- The proposed approach supports non-destructive structural monitoring.

**Abstract:**

The development of advanced high-strength steels (AHSS) for the automotive industry requires optimizing the balance between mechanical properties and durability in aggressive environments. This study investigates the effects of two heat treatment routes on the microstructure and corrosion resistance of a transformation-induced plasticity (TRIP) steel (Fe-0.2C-1.75Mn-0.5Si-1Al). Route A includes a full austenitizing step at 1000 °C prior to intercritical annealing, whereas Route B omits this step and begins directly with intercritical annealing at 800 °C. Microstructural characterization (SEM/XRD), electrochemical assays, and eddy current tests were employed. The results revealed that Route A yields a homogeneous microstructure with 12.7% retained austenite, higher than the 7.7% obtained with Route B. Electrochemically, the steel from Route A exhibited the greatest resistance, with the lowest corrosion current density (i_corr_) of 3.72 µA/cm^2^ and a more noble corrosion potential (E_corr_) of −743 mV compared to SCE. The improvement mechanism is that the homogeneity induced by complete austenitization minimizes the formation of internal galvanic cells between phases; likewise, the higher austenite fraction provides superior chemical stability, which favors denser passivation. Finally, Route A exhibited the lowest loss of electrical conductivity (16%), validating the use of eddy currents for monitoring the integrity of advanced steels.

## 1. Introduction

Nowadays, the development of novel materials is a key task for technologists and scientists engaged in improving the characteristics and performance of products. The engineering, designing, and manufacturing of improved structural materials for diverse industries (automotive, aerospace, agriculture, among others) all require enhancing mechanical properties and technological capabilities, as well as minimizing the weight of components and improving corrosion resistance. In the automotive industry particularly, these attributes and characteristics enhance the functionality of materials, offering better performance to components and vehicles.

The use of structural materials, however, involves high costs. Hence, proposing and developing lower-cost materials without diminishing their functionality is a crucial requirement [1,2,3,4,5]. Microalloyed steel and advanced high-strength steels (AHSS) are the most used materials in the automotive sector. Recently, high-strength low-alloy steels (HSLA) have also been widely studied, as some components and structures in transportation and vehicles (frame and chassis) supports considerable loads and are subjected to severe environmental conditions. Innovation has led to manufacturing new alloys for these applications, such as AHSS; specifically, in automotive structural components. The AHSS include Transformation-Induced Plasticity (TRIP) steels which are developed for the bodies of lightweight vehicles. A significant characteristic of TRIP steels is the influence of alloying elements, such as silicon, manganese, aluminum, vanadium, etc., on their microstructures and phase transformations during heat treatment processes [6,7,8,9]. These elements control phase transformations and stabilize retained austenite, resulting in steels with higher strength, greater ductility, and improved deformation behavior. The interaction between chemical composition, microstructure, and phase distribution is fundamental to the mechanical performance of TRIP steels [10,11].

TRIP steels possess a peculiar microstructure consisting of austenite with a certain thermodynamic instability that achieves transformation to martensite during material loading or deformation. Additionally, the presence of residual austenite within a ferrite matrix is observed in many TRIP steels for the automotive industry. Such steels may also contain hard phases of bainite and martensite. The bainitic transformation that occurs in TRIP steels has peculiarities related to the influence on austenite retention which occurs at room temperature. This phenomenon is associated with the alloying elements, the austenite grain size, and the prior deformation of austenite [12,13]. TRIP steels improve the strength of the low-alloy material because its microstructure has at least 5% retained austenite which, when deformed, is transformed into martensite. This phase is the main component in improving the performance of this property. Such a microstructure is formed after intercritical annealing and subsequent isothermal annealing in the transformation region, a process called austenitizing, which results from the suppression of carbide formation during transformation due to the presence of Al and Si [10,14,15].

Recent studies have discussed the role of microstructure in the corrosion resistance of diverse common alloys and steels. However, investigations into the chemistry, microstructure and electrochemical behavior of new developments of AHSS are still under further consideration, and the background literature on these topics is limited. For instance, recent investigations suggest that TRIP effect, even if it contributes to advantages in mechanical performance, may also affect corrosion behavior by modifying the local electrochemical environment through phase transformation [12,16,17]. Sarkar et al. [12] reported experimental values for the corrosion potential and current density of multiphase steel in NaCl solution. The results obtained indicated that the amount of martensite increases corrosion susceptibility compared to a ferrite-pearlite steel, where heat treatment cycles can generate a desired microstructure, leading to mechanical and corrosion properties superior to those of conventional steels. Contrarily, an investigation performed by Dong et al., showed that as tempering time was increased, the proportion of islands of martensite–austenite was reduced, resulting in a decrease in the number of micro-galvanic couples, leading to a decrease in the corrosion current density. Additionally, it was observed that uniform distribution of ferrite and residual austenite can hinder the diffusion of corrosion products, inhibiting the dissolution rate of the analyzed specimens, thereby enhancing the corrosion resistance [16].

It has been observed that the production of martensite in the material may have a negative effect on corrosion resistance, highlighting the importance of evaluating its impact under specific conditions. In this regard, non-destructive testing (NDT) may be seen as an important microstructural technique that lets us detect microstructural changes in materials subjected to corrosion environments. This technique has not been widely used to predict catastrophic failures in materials subjected to corrosion conditions.

In this study, we will investigate and analyze the corrosion resistance of a multiphase steel with TRIP effect, ensuring a balance between its mechanical properties and its durability in marine environments. Therefore, non-destructive testing (NDT) plays a key role in the evaluation of the integrity of this type of steel.

## 2. Materials and Experimental Procedure

### 2.1. TRIP Steel Production

TRIP steel with a chemical composition of 0.20% C, 1.00% Al, 1.69% Mn, and 0.47% Si was produced in an electric induction vacuum furnace from AISI-1020 steel to which Si, Mn, and Al were added as alloying elements. The steel ingot obtained from the melt was hot-rolled at 1200 °C with a 65% reduction to produce a 13 mm thick plate, from which test specimens were prepared for corrosion and non-destructive testing.

### 2.2. Heat Treatment for TRIP Steel Production

**Figure 1:**
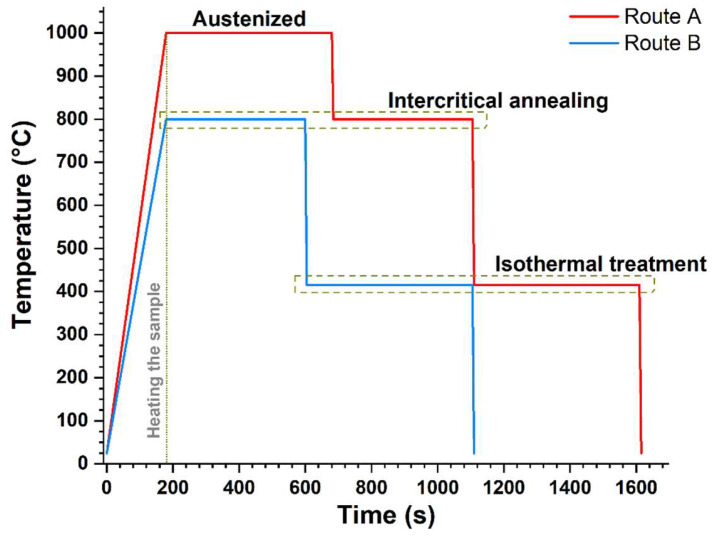
Diagram for the heat treatment routes applied to steel.

Figure 1 shows the two heat treatment (HT) routes (Route A and Route B) used to obtain specimens with the characteristic phases of a TRIP steel. Route A (red line) was subjected to isothermal treatment in a salt bath at 415 °C for 500 s. Finally, it was quenched in water. Through this HT route, a microstructure was formed, in which ferrite encapsulates retained austenite and bainite. This is because complete austenitization promotes carbon dissolution in iron, allowing ferrite to form during intercritical annealing at the grain boundaries of unstable austenite [18,19,20]. This austenite is enriched in carbon during the bainitic transformation that takes place during isothermal salt treatment. In this process, the capacity to retain metastable austenite at room temperature is greater, due to the smaller grain size and higher carbon content [21]. For Route B, the steel was water-quenching. Through this HT route, the formation of retained austenite and bainite is promoted at ferrite grain boundaries, generating a ferrite microstructure encapsulated by retained austenite and bainite. In both HT routes, the objective of the isothermal treatment is to stabilize the austenite by enriching it with carbon during the bainitic transformation [22]. In low-carbon steels, the bainitic transformation temperature is 350 to 500 °C. However, it is recommended that it be performed at approximately 400 °C [21,22,23], with residence times between 120 and 500 s, to avoid complete austenite transformation and carbide precipitation [24].

### 2.3. Microstructural Characterization of Specimens

For microstructural characterization of the specimens, a JSM-7600F field-emission scanning electron microscope equipped with a Bruker XFlash 6/30 EDS detector was used. Austenite identification was performed by X-ray diffraction using a Bruker XRD diffractometer with a copper tube. The quantification phases were determined by Rietveld analysis using the Materials Analysis Using Diffraction (MAUD) software [25]. Metallographic preparation was carried out by grinding with SiC abrasive paper (#180–#2000), followed by polishing with 3 and 0.1 μm diamond pastes. The microstructure was then revealed by chemical etching with 2% Nital.

### 2.4. Corrosion and Non-Destructive Testing

For the corrosion test, the samples were immersed in synthetic seawater [26], and a three-electrode cell was used with a saturated calomel electrode (SCE) as the reference and a platinum wire as the auxiliary electrode. Potentiodynamic measurements were performed with a potential sweep from −500 to 2000 mV against the open-circuit potential (OCP), at a scan rate of 1.0 mV/s. Electrochemical impedance spectroscopy (EIS) measurements were performed over the frequency range of 100 kHz to 10 mHz, with an amplitude of ±15 mV across the OCP.

## 3. Results and Discussion

### 3.1. Microstructural Characterization

**Figure 2:**
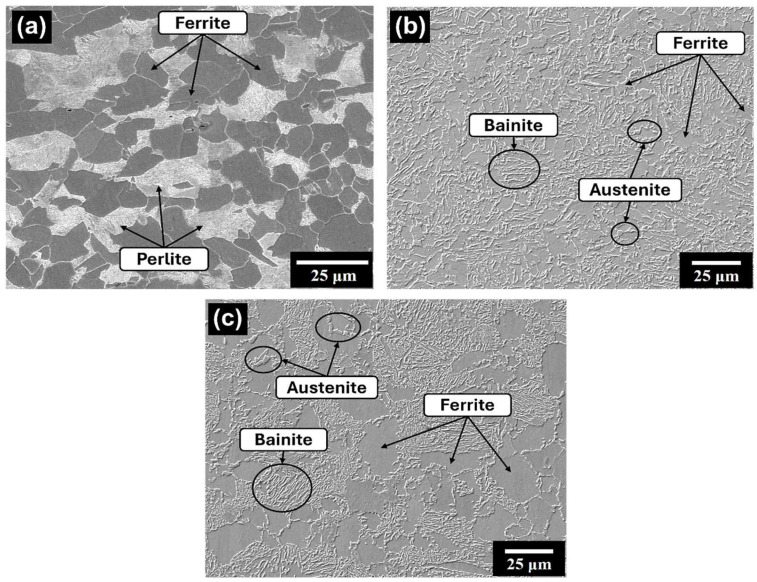
SEM micrographs of (**a**) MB, (**b**) Steel A, and (**c**) Steel B.

Figure 2 shows the microstructures of untreated steel (MB), as well as those of steel heat-treated by Route A (Steel A) and Route B (Steel B). The untreated steel has a microstructure (Figure 2a) composed of ferrite (dark areas) and pearlite (light areas). This morphology results from slow cooling after hot rolling and is characteristic of low-carbon steels [1,3,26]. Figure 2b shows the microstructure of Steel A, composed of a ferrite matrix, with bainite and retained austenite present within it. The morphology of this microstructure is a product of complete austenitization; this is because, as ferrite grows at the grain boundaries, the bainite and retained austenite remain inside the ferritic matrix. Conversely, in the microstructure of Steel B (Figure 2c), the intercritical treatment promotes the presence of ferrite and at the grain boundaries the formation of austenite, and during the bainitic transformation, part of the austenite is retained in a metastable manner due to the enrichment with carbon, resulting in a morphology where the ferrite is surrounded by bainite and retained austenite.

**Figure 3:**
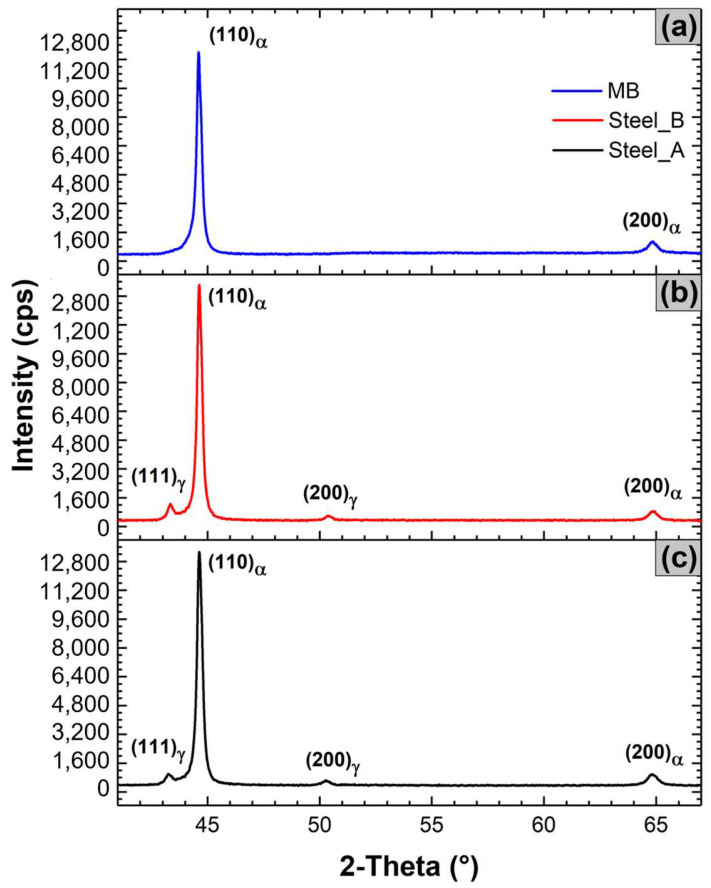
Diffractograms of steel. (**a**) MB, (**b**) Steel B, and (**c**) Steel A.

Figure 3 shows the diffractograms obtained for the steel under its three conditions (MB, Steel A, and Steel B). As shown in Figure 3a, the diffraction peaks in the (110) and (200) planes correspond to ferrite. As mentioned in Figure 2a, this steel is ferritic-pearlitic, so no other phases are observed. Figure 3b shows the diffractogram for Steel B, which reveals the presence of austenite in addition to ferrite, with principal planes (111) and (200). The diffractogram for Steel A (Figure 3c) shows the same reflections from the principal planes of the ferrite and austenite phases as in Steel B, thus demonstrating the presence of ferrite, bainite, and retained austenite in both steels, with different morphologies due to different heat treatment routes. Quantitative Rietveld analysis determined that Steel A contains 87.3 wt.% ferrite and 12.7 wt.% austenite, while steel B contains 92.3 wt.% of ferrite and 7.7 wt.% of austenite. These retained austenite fractions are within the range typically reported for low-carbon TRIP steels subjected to intercritical annealing and bainitic transformation treatments [21,24]. Similar phase fractions have also been quantified by X-ray diffraction in TRIP-assisted steels, confirming the suitability of this technique for retained austenite determination [10]. The higher retained austenite content obtained in Steel A is attributed to the complete austenitization route, which promotes a more uniform redistribution of carbon prior to bainitic transformation, thereby enhancing austenite stabilization during cooling. In contrast, the intercritical treatment applied to Steel B resulted in a lower retained austenite fraction and a more heterogeneous phase arrangement. Similar trends have been reported by Zurnadzhy et al. [8] and Wiewiórowska et al. [9], who demonstrated that heat treatment parameters strongly influenced phase balance and retained austenite stability in TRIP-assisted steels. Furthermore, the microstructural differences between Steel A and Steel B extend beyond phase fractions alone. Recent studies have shown that the mechanical and thermodynamic stability of retained austenite is strongly governed by the nature and distribution of the surrounding phases [14,27]. In Steel A, bainite and retained austenite are dispersed within a continuous ferritic matrix, resulting in a more homogeneous microstructure. Conversely, Steel B exhibits ferrite regions surrounded by harder constituents, generating a more heterogeneous phase distribution. Such differences in phase arrangement can influence local strain partitioning, retained austenite stability, and overall performance in TRIP steels. Therefore, the superior behavior observed in Steel A cannot be attributed solely to its higher retained austenite fraction, but also to the more favorable spatial distribution of hard and soft phases produced by the austenitizing route.

### 3.2. Corrosion Assessment

**Figure 4:**
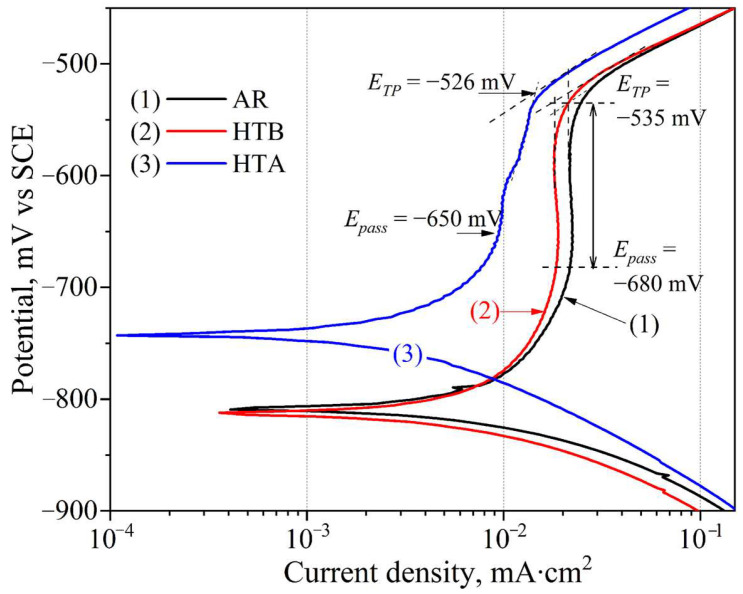
Potentiodynamic curves for samples in the synthetic seawater at room temperature. The passivation potential ranges are shown.

Figure 4 shows the polarization curves for the steel under the three conditions. The corrosion potentials (E_corr_) in steel MB and steel B were similar, at approximately −810 mV and −813 mV vs. SCE, respectively, with corrosion current densities (i_corr_) of 8.1 and 7.0 µA/cm^2^, respectively. The effect of the austenitizing heat treatment (steel A) is evident, resulting in a shift in E_corr_ to more positive values (−743 mV vs. SCE) and a reduction of i_corr_ to 3.72 µA/cm^2^. The Tafel parameters were obtained from potentiodynamic measurements using the intersection method [28].

Additionally, the polarization resistance (R_p_) was calculated using the Stern–Geary relationship from the anodic and cathodic Tafel slopes and the corrosion current density [29]. The obtained R_p_ values were 18.55 Ω·cm^2^, 16.70 Ω·cm^2^, and 16.03 Ω·cm^2^ for Steel A, Steel B, and base metal, respectively. The higher R_p_ value obtained for Steel A indicates greater resistance to charge transfer processes and improved corrosion resistance. The base metal exhibited the lowest R_p_ value, confirming its higher corrosion susceptibility. These results are consistent with the lower corrosion current density observed for Steel A and the superior electrochemical performance discussed in the EIS analysis.

All three steel samples exhibited a passivation region (limiting current density) following the activation region. This behavior is attributed to the formation of iron hydroxides [30]. The passivity of iron in alkaline solutions is due to the formation of hydrated ferrous oxide (FeO·H_2_O) or ferrous hydroxide (Fe (OH)_2_) phases [16], which decompose when these phases become thermodynamically unstable. After the passivation stage, the potentiodynamic curves revealed anodic dissolution, which continued until the end of the overpotential region.

The passivation region is associated with the formation of a surface layer composed primarily of iron hydroxides and hydrated iron oxides, which act as a temporary barrier to anodic dissolution. According to Pourbaix [30], these corrosion products can reduce the active dissolution rate by partially isolating the metallic substrate from the electrolyte. However, in chloride-containing environments such as artificial seawater, the stability of these passive films is limited. Chloride ions may penetrate and locally destabilize the corrosion layer, promoting its dissolution and leading to renewed anodic activity at higher overpotentials. Similar behavior has been reported for low-carbon and TRIP-assisted steels exposed to saline environments [12,16].

**Figure 5:**
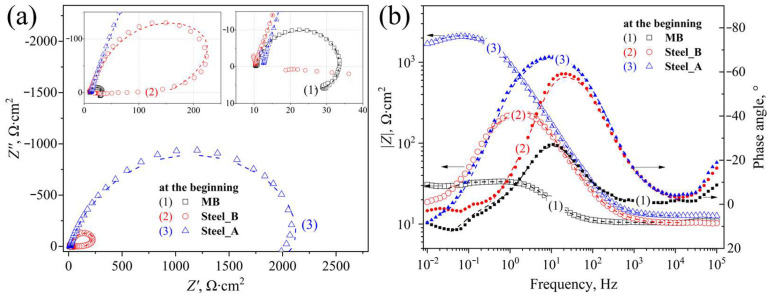
(**a**,**b**) EIS measurements of the samples at the beginning of immersion. Scatter and dashed curves represent experimental data and the ECM fit, respectively.

**Figure 6:**
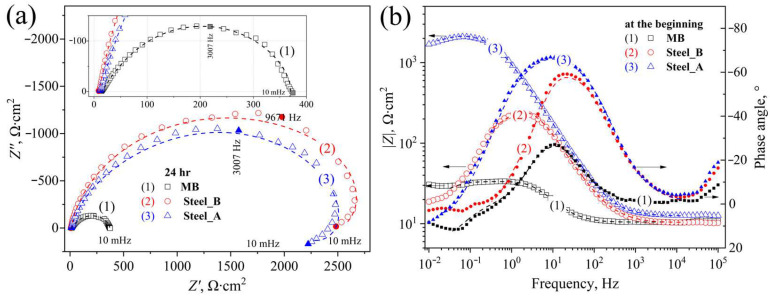
(**a**,**b**) EIS measurements of the samples after 24 h of immersion. Scatter and dashed curves represent experimental data and the ECM fit, respectively.

The results of EIS measurements for the MB, Steel B, and Steel A samples at the beginning and after 24 h of immersion in artificial seawater are shown in Figure 5 and Figure 6, respectively. At both immersion times, the MB sample exhibits the lowest impedance modulus (|Z|), approximately 30 Ω· cm^2^ at 10 mHz. In contrast, the Steel A sample presents the highest |Z| at the start of immersion, approximately 1694 Ω·cm^2^ (Figure 5b). After 24 h, both Steel B and Steel A samples show similarly high impedance values, approximately 2480 and 2230 Ω·cm^2^, respectively (Figure 6b). At both times, inductive behavior is observed at lower frequencies (Figure 5a and Figure 6a); this is attributed to adsorption processes involving chemical species and intermediate electrochemical reactions of oxides. These mechanisms contribute to the formation of a corrosion product layer that evolves toward more stable states, resulting in increased impedance at the end of the immersion period.

The shapes of the Nyquist plots and the amplitudes of the Bode phase angle plateaus at mid-frequencies suggest a combination of corrosion mechanisms. This is attributed to interfacial capacitance contributions from the corrosion product layer—on one side facing the electrolyte, and on the other interfacing with the metal substrate.

**Figure 7:**
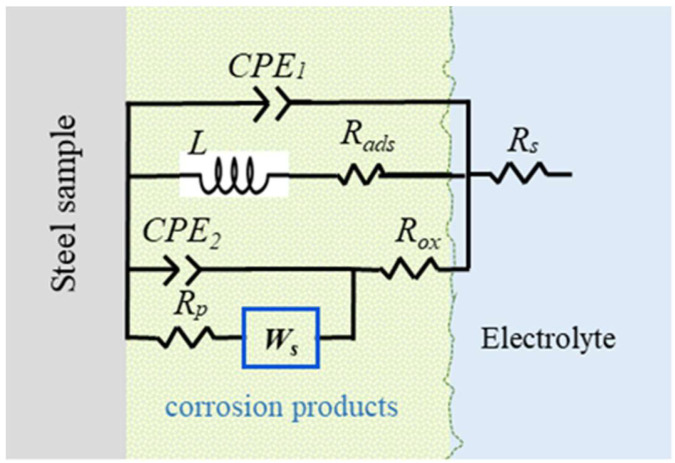
Analog Equivalent Circuit Model ECM.

The equivalent circuit model (ECM) shown in Figure 7 represents an electrical analog of the dominant corrosion mechanisms. It includes the electrolyte resistance (R_s_) in series with a parallel network comprising the following: CPE_1_ (representing the double-layer capacitance), a resistive–inductive pair for adsorption effects (R_ads_ and L), and an oxide resistance (R_ox_), and CPE_2_/(W_s_−R_p_) combination modeling the double layer at the metal/corrosion product interface [31].

**Figure 8:**
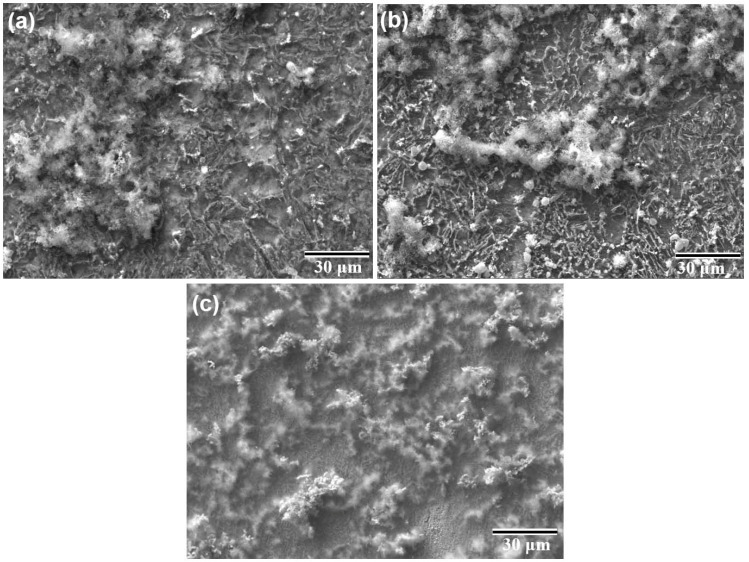
(**a**–**c**) SEM images of the corroded surface of samples in artificial seawater: (**a**) MB, (**b**) Steel B, and (**c**) Steel A.

The formation of corrosion products after 24 h of immersion is shown in Figure 8. The SEM image of the corroded surface of sample MB reveals a non-homogeneous corrosion layer consisting of porous structures and residual salts from the artificial seawater solution (Figure 8a), visible as surface deposits, primarily MgCl_2_·6H_2_O and Na_2_SO_4_, or their dissociation compounds in water [26]. For Fe-0.2C-1.75Mn-0.5Si-1Al steel, the expected corrosion products after immersion are primarily iron hydroxides, Fe(OH)_2_ and Fe(OH)_3_, which serve as precursors to iron oxide (Fe_2_O_3_) [18]. Minor formation of other oxides, such as Mn_2_O_3_ and the mixed oxide Fe(Mn,Al)_2_O_4_, are also expected in low concentrations. Similarly, samples Steel B and Steel A exhibited comparable corrosion products (Figure 8b,c). However, the morphology of the layer adjacent to the metal surface in these samples is denser and more uniform. This observation is consistent with the higher impedance modulus values of approximately 2480 and 2230 Ω·cm^2^ (Figure 6b), suggesting increased corrosion resistance due to the formation of a more stable corrosion product layer.

**Figure 9:**
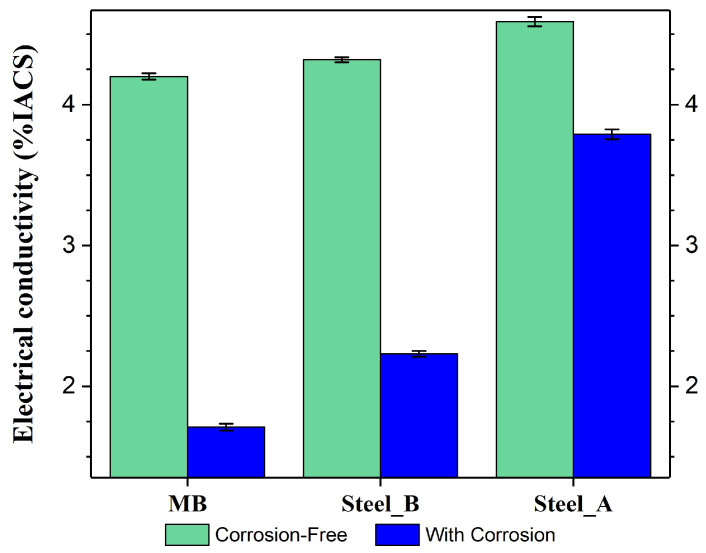
Electrical conductivity of MB, Steel B, and Steel A.

In Figure 9, after the corrosion test Steel A shows a higher initial conductivity, equivalent to 4.59% IACS, with a conductivity loss of 16.01%; this is due to its microstructure with lower ferrite content and higher austenite content. The controlled presence of austenite benefits the chemical stability of the surface because its FCC crystalline structure has higher atomic density and higher corrosion resistance compared to ferrite with BCC crystalline structure, favoring surface passivation on aggressive surfaces [1,2,3]. In comparison with Steel B, which has a higher percentage of ferrite and a lower percentage of retained austenite, there is an average loss of conductivity of 48.26%. These results agree with the Rietveld analysis shown in microstructural characterization section. Furthermore, the differences between Steel A and Steel B cannot be explained solely by the retained austenite fraction. Although both heat treatments promoted the formation of retained austenite, the austenitizing route produced a more homogeneous microstructure. In Steel A, the harder constituents were dispersed within a continuous ferritic matrix, whereas the intercritical route generated a heterogeneous distribution of hard and soft phases. According to [14], the stability and effectiveness of retained austenite strongly depend on the adjacent phases. Consequently, the microstructural arrangement obtained in Steel A reduced local electrochemical heterogeneities and limited galvanic interactions, resulting in lower conductivity degradation after corrosion exposure.

However, even with the presence of austenite, the inhomogeneous phase distribution produced by the intercritical heat treatment is not as favorable as in Steel A. This heterogeneity could generate internal galvanic zones between ferrite (less noble) and austenite (more noble), thus promoting differential degradation and a significant loss of conductivity [6,32,33]. In the MB, a higher conductivity decrease is observed at 59%, indicating a degradation in the electron pathways of electron interaction. This behavior is attributed to the high susceptibility of ferrite to oxidation, and also to the presence of pearlite, which includes cementite (Fe_3_C), an electrically insulating phase that disrupts electronic conduction pathways. In addition, the larger grain size (18.4 μm) reduces the grain boundary density, which decreases the number of barriers to corrosion propagation and favors the formation of internal galvanic cells. The high proportion of ferrite contributes significantly to widespread oxidation, given its lower chemical resistance against corrosive media [27,33]. Phase composition, carbon content, and grain size are key factors in determining the electrochemical behavior of the studied steels. The controlled presence of austenite and a fine, homogeneous microstructure enhances both conductivity and corrosion resistance. In contrast, a higher proportion of ferrite and cementite, accompanied by a coarse microstructure, tends to impair electrochemical performance.

## 4. Conclusions

The microstructural design of advanced Fe-0.2C-1.75Mn-0.5Si-1Al steel, through specific heat treatment routes, defines its electrochemical resilience in aggressive marine environments. The variation in phase content, particularly on retained austenite, directly influences corrosion resistance, hardness, and electrical conductivity. This study demonstrates that implementing a full austenitization stage (Route A) constitutes optimal treatment, overcoming the limitations of direct intercritical annealing (Route B). The mechanisms underlying this behavior are summarized as follows:

The thermal homogenization of Route A induces grain refinement to 12.8 µm and maximizes the retained austenite fraction to 12.7% (a relative increase of ~65% compared to Route B). This microstructural architecture suppresses the formation of internal galvanic microcells and promotes the development of a highly compact passivating layer, based on the high atomic density and intrinsic chemical stability of the austenite’s FCC structure. As a direct result of phase stability, steel processed by Route A reduces corrosion current density (i_corr_) by 54% compared to the base metal and by ~47% compared to Route B, thus solidifying its viability for applications under highly chloride-aggressive conditions.

From a solid-state physics perspective, the superior surface chemical integrity of Route A drastically mitigates electron scattering induced by impurities and corrosion products. This allows for exceptional electronic continuity, limiting conductivity loss to only 16%, in stark contrast to the critical degradation observed on Route B (48%) and in the base metal (59%).

The phenomenological correlation between electrical conductivity retention and resistance to localized degradation conclusively validates the eddy current technique as a robust predictive tool. It offers a viable technical solution, not only for extending the service life and operational safety of advanced steel components, but also to optimize their maintenance costs through high-precision non-destructive diagnostics.

## Data Availability

The authors confirm that the data supporting the findings of this study are available in the article and its complementary materials.

## References

[1] Rodríguez-Muñoz J.L., Pacheco-Cedeño J.S., Bedolla-Jacuinde A., Medina-Flores A. (2023). Effect of microstructural morphology in low-carbon TRIP steels on their mechanical properties. MRS Adv..

[2] García Aguirre K.A. (2020). Procesado y Caracterización de Propiedades Mecánicas de Acero TWIP Mediante Técnicas Pulvimetalúrgicas. Ph.D. Dissertation.

[3] Fonstein N. (2015). Advanced high strength sheet steels: Physical metallurgy, design, processing, and properties. Advanced High Strength Sheet Steels: Physical Metallurgy, Design, Processing, and Properties.

[4] Salinas A., Artigas A., Perez-Ipiña J., Castro-Cerda F., Garza-Montes-De-Oca N., Colás R., Petrov R., Monsalve A. (2018). Effects of Heat Treatment on Morphology, Texture, and Mechanical Properties of a MnSiAl Multiphase Steel with TRIP Behavior. Metals.

[5] Li G.Q., Shen Y.F., Jia N., Feng X., Xue W. (2022). Microstructural evolution and mechanical properties of a micro-alloyed low-density δ-TRIP steel. Mater. Sci. Eng. A.

[6] Bleck W., Guo X., Ma Y. (2017). The TRIP Effect and Its Application in Cold Formable Sheet Steels. Steel Res. Int..

[7] Oja O., Saastamoinen A., Patnamsetty M., Honkanen M., Peura P., Järvenpää M. (2019). Microstructure and Mechanical Properties of Nb and V Microalloyed TRIP-Assisted Steels. Metals.

[8] Zurnadzhy V., Efremenko V., Petryshynets I., Dabalà M., Franceschi M., Wu K., Kováč F., Chabak Y., Puchy V., Brykov M. (2022). Alternative Approach for the Intercritical Annealing of (Cr, Mo, V)-Alloyed TRIP-Assisted Steel before Austempering. Metals.

[9] Wiewiórowska S., Siemiński M., Śleboda T., Łukaszek-Sołek A., Dyl T., Koczurkiewicz B. (2022). Determination of Two-Stage Heat Treatment Parameters in Industrial Conditions in Order to Obtain a TRIP Structure in Low-Alloy Carbon Steel Wires. Materials.

[10] Zhao L., Van Dijk N.H., Brück E., Sietsma J., van der Zwaag S. (2001). Magnetic and X-ray diffraction measurements for the determination of retained austenite in TRIP steels. Mater. Sci. Eng. A.

[11] Gorlenko D.A., Konstantinov D.V., Polyakova M.A., Dabalá M. (2022). TRIP steels: The features of chemical composition and structure, prospects of application (overview). CIS Iron Steel Rev..

[12] Sarkar P.P., Kumar P., Manna M.K., Chakraborti P.C. (2005). Microstructural influence on the electrochemical corrosion behaviour of dual-phase steels in 3.5% NaCl solution. Mater. Lett..

[13] Mehranpour M.S., Sohrabi M.J., Jalali A., Kalhor A., Heydarinia A., Aghdam M.Z., Mirzadeh H., Malekan M., Shahmir H., Rodak K. (2025). Coupling different strengthening mechanisms with transformation-induced plasticity (TRIP) effect in advanced high-entropy alloys: A comprehensive review. Mater. Sci. Eng. A.

[14] Wu S., Mao W., Zhao L., Hu Z., Ma W., Sheng S., Wang Q. (2026). Adjacent phase-governed stability of retained austenite and hetero-deformation-induced hardening in TRIP-assisted steels. Int. J. Miner. Metall. Mater..

[15] Avishan B., Charchi Aghdam M., Hosseinzadeh Khanmiri M. (2025). Mechanical Stability of High-Carbon Retained Austenite and Corresponding TRIP Effect in Austempered Ductile Iron (ADI). Int. J. Met..

[16] Dong X.X., Shen Y.F. (2022). Improving mechanical properties and corrosion resistance of 0.5 wt.% C TRIP steel by adjusting retained austenite stability and microstructural constituents. Mater. Sci. Eng. A.

[17] Cheng Y., Zhang X., Zheng Y.-F., Wan X.-L., Zhao T.-L., Cheng L., Liu J., Wu K.-M. (2026). Exploring modulation of TRIP effects on localized corrosion behavior of medium-manganese steel. Corros. Commun..

[18] Jacques P.J. (2004). Transformation-induced plasticity for high strength formable steels. Curr. Opin. Solid State Mater. Sci..

[19] Grajcar A., Skowronek A., Radwański K. (2022). Mechanical behavior and stability of dispersed retained austenite in thermomechanically rolled and isothermally-treated TRIP-aided multiphase steel. Mater. Sci. Eng. A.

[20] Kozłowska A., Grajcar A., Radwański K., Opara J., Matus K., Nuckowski P.M. (2022). Microstructure and temperature-dependent mechanical behavior of hot-rolled TRIP-assisted microalloyed steel. Mater. Charact..

[21] Sakuma Y., Matsumura O., Takechi H. (1991). Mechanical properties and retained austenite in intercritically heat-treated bainite-transformed steel and their variation with Si and Mn additions. Metall. Trans. A.

[22] Bleck W., Ohlert J., Papamantellos K. (1999). Sheet metal forming behaviour and mechanical properties of TRIP steels. Steel Res..

[23] Tsukataimi I., Hashimoto S., Inoue T. (1991). Effects of Silicon and Manganese Addition on Mechanical Properties of High-strength Hot-rolled Sheet Steel Containing Retained Austenite. ISIJ Int..

[24] Matsumura O., Sakuma Y., Takechi H. (1992). Retained Austenite in 0.4C-Si-1.2Mn Steel Sheet Intercritically Heated and Austempered. ISIJ Int..

[25] Lutterotti L., Kolb U., Shankland K., Meshi L., David W.I.F. (2012). Quantitative Phase Analysis: Method Developments. Uniting Electron Crystallography and Powder Diffraction.

[26] (2021). Standard Practice for the Preparation of Substitute Ocean Water.

[27] Talapatra A., Datta J., Bandhyopadhyay N.R. (2013). Structure-Properties Relationship of TRIP-assisted Steels by Non-destructive Testing Method. Chem. Mater. Eng. (CEASE Publ.).

[28] (2004). Standard Practice for Calculation of Corrosion Rates and Related Information from Electrochemical Measurements.

[29] Yang F., Yang H., Fu W., Li X., Gao Y., Qiao Z., Zhuo Z. (2026). Corrosion and discharge mechanism of Mg-Ga-Hg anode for magnesium-air batteries. J. Power Sources.

[30] Pourbaix M. (1966). Atlas of Electrochemical Equilibria in Aqueous Solutions.

[31] Orazem M.E., Tribollet B. (2008). Electrochemical Impedance Spectroscopy.

[32] Yesith Mendoza González E., Amparo Quintero Ortiz L., Santos Castañeda Ingeniería Metalúrgico G. (2010). Ensayos no destructivos como herramienta para el dimensionamiento de discontinuidades en la superficie externa de tuberías. Rev. UIS Ing..

[33] Gu X., Xu Y., Peng F., Misra R., Wang Y. (2019). Role of martensite/austenite constituents in novel ultra-high strength TRIP-assisted steels subjected to non-isothermal annealing. Mater. Sci. Eng. A.

